# Recruitment, experience, and retention among women with HIV and hazardous drinking participating in a clinical trial

**DOI:** 10.1186/s12889-020-09233-z

**Published:** 2020-07-27

**Authors:** Shantrel S. Canidate, Christa L. Cook, Deepthi Varma, Giselle D. Carnaby, Nicole Ennis, Nichole E. Stetten, Robert L. Cook

**Affiliations:** 1 University of Florida, College of Public Health and Health Professions, 2004 Mowry Road PO Box 100231, Gainesville, FL 32610-0182 USA; 2 College of Medicine, University of Florida, Gainesville, FL 32610 USA; 3 College of Nursing, University of Central Florida, Orlando, FL 32816 USA; 4 College of Health Professions and Sciences, University of Central Florida, Orlando, FL 32816 USA; 5 College of Medicine, Florida State University, Tallahassee, FL 32304 USA

**Keywords:** Women, Alcohol, Participation, HIV, Clinical research, Hazardous drinking

## Abstract

**Background:**

Despite efforts by the NIH to enhance the participation of women and minorities in clinical research, women with HIV continue to remain underrepresented in alcohol intervention research. The purpose of this study is to better understand the reasons why women with HIV and hazardous drinking participated in the WHAT-IF? study and to discuss their experience (positive or negative) in the study. The WHAT-IF? study was a randomized clinical trial that evaluated pharmacotherapy for a reduction in drinking among women with HIV.

**Methods:**

Convenience and theoretical sampling were used to recruit women with HIV and hazardous drinking to complete qualitative interviews. These women had previously completed a clinical alcohol intervention trial and had consented to be contacted in the future for study-related purposes. The biopsychosocial model was used to frame the interview questions that assessed multiple determinants of drinking behavior and helped explain linkages to broader health constructs.

**Results:**

A total of 20 women with HIV and hazardous drinking completed the qualitative interview. Several factors were identified by the women as influential in their decision to participate in the WHAT-IF? study, such as the ability to quit or reduce their drinking to nonhazardous levels (biological), the ability to gain knowledge or a greater understanding of the negative effects of hazardous drinking on HIV disease progression (psychological), and peer pressure and monetary compensation (social). Also, the women identified factors (positive or negative) associated with their clinical trial experience, such as the effects of the study medication on the woman’s body (biological), thoughts and feelings toward study procedures (i.e. medication, lab work, study assessments) and the length of the study (psychological), and the interactions with the WHAT-IF? study staff (social).

**Conclusion:**

Recruiting and retaining women with HIV in alcohol intervention research remains a challenge. Findings from this study suggest that women with HIV who are hazardous drinkers may benefit from participating in research studies that could help them to reduce or quit their drinking, increase their knowledge about specific behavior changes, and earn monetary compensation. Also, positive staff interactions may be instrumental in retaining minority women in alcohol intervention research.

## Background

Despite efforts by the National Institute of Health (NIH) to enhance the participation of women and minorities in clinical research, women with HIV continue to remain underrepresented in alcohol intervention research [5, 6, 29, 30]. In substance use research, researchers have failed to adequately recruit women, and frequently made the mistake of generalizing results to women without analyzing sex-specific differences [4, 17, 25, 36, 44]. The results of most studies examining pharmacotherapy and behavioral interventions for alcohol use disorders (AUD) are not generalizable to women due to the under-sampling of women in those studies [17, 31, 33, 45].

A growing body of literature has evaluated gender disparities in treatment-seeking patterns among men and women in alcohol intervention research [15, 24, 25, 36, 44]. Importantly, in these studies, women who displayed positive attitudes toward treatment-seeking practices and perceived the benefits of participating in the study were found to be more likely to participate in alcohol intervention research. Previous research has cited reasons for treatment entry among women varied based on the women’s lifetime general treatment, age, race/ethnicity, employment rate, and family history of alcoholism [11, 17, 21, 39]. Likewise, women with HIV may demonstrate certain attitudes toward treatment-seeking practices, such as perceiving a need for substance use treatment, having less knowledge about the benefits of treatment options, or negative beliefs about treatment [21]. On the contrary, well-documented barriers to treatment-seeking practices among women include lack of services for pregnant women and preferring one form of treatment of intervention over the other [19, 22, 23, 31–33].

Moreover, little is known about the clinical trial experiences of women with HIV who participate in alcohol research. Our ability to understand these processes, from women’s perspectives could enhance effective treatment approaches [20, 35, 41]. Women participating in a qualitative study examining their experiences of managing depressive symptoms reported the personal qualities of healthcare professionals were associated with their willingness to discuss sensitive topics [41]. Additional qualitative studies examining study retention reported the interpersonal skills of the healthcare professionals are instrumental in enrolling, retaining, and improving intervention efficacy [1, 26, 41].

The purpose of this study is to better understand the reasons why women with HIV and hazardous drinking participated in the WHAT-IF? study and to discuss their experience (positive or negative) in the study. Conducting qualitative research (particularly in underserved, disadvantaged groups) could provide critical data on the perceptions, cultural relevancy, acceptability, and salience of specific aspects of intervention approaches of the randomized clinical trial that are acceptable and not acceptable to these populations [9]. The retention rates in the WHAT-IF? study was above satisfactory, with over 89 and 85% completing the 4-month and 7-month follow-ups respectively [8]. This is notable and does suggest that positive experiences in the study could enhance study retention.

## Methods

### Study design

Qualitative interviews were conducted among women with hazardous drinking and HIV who participated in the WHAT-IF? (Will Having Alcohol Treatment Improve my Functioning) study. The WHAT-IF? study was a large, multi-site double-blind randomized clinical trial that evaluated pharmacotherapy for a reduction in drinking among women with HIV in Miami, FL. Detailed information about the study is published elsewhere [7, 8]. Briefly, the primary goal of the study was a reduction in hazardous drinking, defined as consuming > 7 drinks per week or > 3 drinks in one sitting. Multiple strategies were employed to reduce barriers to recruitment among women into an alcohol intervention trial. The research team placed brochures in clinics and community settings and encouraged current participants to refer to others. Women with HIV were eligible for study participation if they were 18 years or older and met criteria for hazardous drinking. Exclusion criteria included current opiate dependence, elevated blood pressure, liver enzymes, or serum creatinine at the time of study enrollment, currently pregnant, currently taking medication for viral hepatitis, tuberculosis, or alcohol treatment, unable to comprehend English or study materials and procedures, current prognosis of < 1 year to live, or recommendation. Eligible women were randomized to receive either naltrexone 50 mg orally or placebo during the first 4 months and completed follow-up assessments at 2, 4, and 7 months. In total, the study enrolled 196 women, of whom 86% were African American, 16% were married or in a long-term relationship, 10% were employed, and 43% had less than high school education. The participant’s mean age was 48 years. While participants in both groups substantially reduced their drinking overtime, naltrexone was only associated with a greater reduction in drinking (*p* < 0.05) at months one and three. Overall, retention was very good, with over 88 and 85% completing the 4-month and 7-month follow-ups respectively.

### Study population

Convenience and theoretical sampling were used to recruit women with HIV and hazardous drinking to complete the qualitative interviews. These women had previously completed the WHAT-IF? study and had consented to be contacted in the future for study-related purposes. The women were recruited at the time of study completion or up to 2 years after completing the study. The primary goal of the qualitative study was to recruit a diverse range of women with HIV, who reportedly reduced, quit, or had no change in their drinking. While the women were chosen for their drinking status (high or low), the interviewer did not know their drinking status going into each interview. However, toward the end of the qualitative study, the interviewer was made aware of four participants for whom the number of drinks per week was extremely high. The participant ID numbers were shared with the research coordinator in Miami who managed study recruitment and retention. A total of four to six participants were recruited every 2 months until theoretical saturation was achieved, and the participants provided no new information in the interview.

### Ethics, consent, and permissions

The WHAT-IF? study and the qualitative study was approved by IRBs at the University of Florida (Gainesville, FL) and the University of Miami Miller School of Medicine (Miami, FL). Written informed consent was obtained at the Miami site. All participants were informed that the purpose of the qualitative study was to better understand why women with HIV choose to participate in alcohol intervention research.

### Study instrument

The biopsychosocial model [14] was used to interpret the responses to the interview questions that assessed multiple determinants of drinking behavior and helped explain linkages to broader health constructs. The interview questions were developed specifically for this study after extensive discussions with the research study team as well as members of the Qualitative Research Colloquium (QRC) at the University of Florida. The QRC is comprised of experienced qualitative investigators and graduate students who provide support and oversight for qualitative research. The complete list of interview open-ended questions fell into five categories that are listed in Table 1. For this study, the category denoted by an asterisk (*) was analyzed.

**Table 1:** Interview open-ended question guide

I. **Drinking History**	
A. Tell me about your experience with drinking alcohol	
1. At what age did you first start drinking? 2. When you first started drinking, were you a light, moderate, or heavy drinker? 3. Why did you decide to start drinking? a. Traumatic experience, social drinker, stress, anxiety, depression … 4. What was it about alcohol that made you continue to drink? 5. Have you tried to quit drinking? b. Tell me about your previous attempts to quit i. Successful or Unsuccessful ii. Time period that you were able to quit or reduce your drinking iii. Were there any barriers that stopped you from quitting or reducing drinking? II. Study Participation* A. How did you find out about the study? 1. What was it about the study that motivated you to participate? 2. Tell me about your participation in other research studies, if any? B. What benefits do you get out of participating in research? C. Tell me about your experience in this study 1. What were some things that you like about the study? 2. What were some things that you did not like about the study? a. What was difficult? D. Describe your relationship with the study staff 1. What did you like about the study staff? 2. What did you not like about the study staff? III. **Changes as a result of participating in the WHAT-IF study** A. What changed in your life as a result of being in the study? 1. How were you able to quit or reduce drinking? a. If so, why do you believe you were successful in doing so? i. Do you think that the study medication helped to reduce or not reduce your drinking? ii. Do you know if you were taking naltrexone or the placebo? b. If not, why do you believe you were unsuccessful? 2. Besides drinking, what else changed in your life as a result of being in the study? B. What do you think are the barriers (things that keep you from doing something) or the facilitators (things that help you do something) to this treatment that would exist in the real world outside of a research setting? IV. **Support** A. Did you have any support from family, friends, etc.? B. When you think of someone that is being supportive, what are they doing? 1. Before being in the study, who did you receive support from? a. In what ways were they supportive or not supportive of you wanting to quit or reduce drinking? 2. During the study, who did you receive support from? a. In what ways were they supportive or not supportive of you wanting to quit or reduce drinking? 3. After completing the study, who did you receive support from? a. In what ways were they supportive or not supportive of you wanting to quit or reduce drinking? C. How important do you think it is to have support? 1. Who do you think are your best providers of support? a. Family, friends, healthcare providers, etc.? 2. Do you think that support is needed in order to successfully quit or reduce drinking? a. Why or why not?	
V. **Next steps** A. Now that you have completed the study, what would you recommend the research staff do next? 1. What could be done differently in the study? B. If you were going to give advice to women about quitting or reducing their drinking, what would you tell them? C. Additional information: 1. Would you like to add anything else?	

### Procedure

The qualitative interviews were conducted by the first author (S.C.) who received hands-on informed consent and qualitative research training by an experienced qualitative investigator. On the day of the scheduled interviews, participants were provided with written informed consent and were able to ask questions related to the study and seek clarification on matters they did not understand. The WHAT-IF? study principal investigator and the qualitative investigator were available remotely for oversight during the interview to clarify study participants’ questions. The interviews were conducted at the University of Miami Clinical Research Building (CRC) housed at the Behavioral Medical Research Center (BMRC) in Miami, FL. During the interview, each participant was asked to identify factors influencing their drinking behavior. Each interview began with a semi-structured format and was digitally recorded. Also, specific interview questions were adapted for each person following the participant’s lead and as theoretical coding dictated. After the interview, the participant was compensated with a $25 gift card for their time.

### Data analysis

This study is an analysis of data collected from qualitative interviews among women with HIV and hazardous drink who completed the WHAT-IF? study. Each interview lasted approximately 1 h and was transcribed by a University of Florida approved, Health Insurance and Portability and Accountability Act (HIPAA) compliant transcription service provider. The transcribed interviews were uploaded to NVivo 11.0 [37], a qualitative data analysis program. The data was stored on an encrypted computer with password protection. Moreover, data analysis was continuous and began after the first interview was completed.

While this is not a grounded theory study, the findings are adapted analytical methods from grounded theory (e.g. iterative and simultaneous data collection and analysis, theoretical saturation). Also, thematic analysis was employed [3, 46]. In this process, the following steps were involved: 1) immerse oneself in the data, 2) generate initial codes, 3) search for themes, 4) review the themes, 5) define and name the themes, and 6) produce the reports. In the beginning, the transcripts were read numerous times to immerse oneself in the data. Also, ideas, questions, and comments were recorded for future use. Next, the data was organized into categories.

## Results

A total of 20 women with HIV and hazardous drinking completed the qualitative interview. The demographic characteristics of the women in the qualitative study were similar to the women in the WHAT-IF? study. In this study, 85% were African American, 80% were single, 100% of the women were unemployed and 60% had less than high school education. The mean age of women was 49.3 (Table 2).

**Table 2:** Demographic characteristics of participants who completed the qualitative interviews (*N* = 20)

Characteristics	(*N* = 20)
Demographics, No. (%)	
Age range, years	Mean 49.3
Race	
Black/African American	17 (85)
White	2 (10)
Other	1 (5)
Ethnicity	
Hispanic/Latino	1 (5)
Non-Hispanic/Latino	19 (95)
Education Level	
Less than High School	12 (60)
High School Graduate	4 (20)
Some College	4 (20)
Marital Status	
Single	16 (80)
In a relationship	4 (20)

Several factors were identified by the women with HIV as influential in their decision to participate in the WHAT-IF? study. The women also identified factors (positive or negative) associated with their clinical trial experience. These were categorized into three themes: biological, psychological, and social (Table 3).

**Table 3:** Themes within the context of the biopsychosocial model related to reasons for participation, experience, and retention in the WHAT-IF? study

Biopsychosocial Factors	Reasons for study participation	Clinical trial experience (positive or negative) and retention in study
Biological	• To reduce drinking to nonhazardous levels • To quit drinking	• Study medication
Psychological	• To gain knowledge about the negative effects of hazardous drinking on HIV disease progression • The opportunity for self-reflection or self-growth	• Thoughts and feelings toward study procedures (e.g. study medication, lab work, computer-based assessments) • Length of study
Social	• To feel pressured by members of social network • To earn supplemental income	• Interactions with research study staff

### Reasons for participating in the WHAT-IF? Study

#### Biological

Among the women, many believed that by participating in the study, they would be able to reduce their drinking to nonhazardous levels or quit altogether.

*“I started drinking every day, and then sometimes I would drink too much the night before, like six beers or something, and in the morning, I’d need a beer badly … but I joined this study because I was interested in helping myself slow down.” (female, 50-60 years old, non-Hispanic White, single)*

*“It’s (clinical trial participation) supposed to help me stop or at least slack up. It’s supposed to help me to stop drinking. At the time, I was all for it, something that’s going to help me stop drinking.” (female, 30-40 years old, non-Hispanic Black/African American, single)*

#### Psychological

For many of the women, participating in the WHAT-IF? study was the first step to changing their lives. Among the psychological factors that influenced participation in the study, many of the women discussed their ability to gain knowledge or a greater understanding of the negative effects of hazardous drinking on HIV disease progression.

*“I knew I would get information to help me slow down on my drinking, at least quit my drinking or slow down on it … ” (female, 50-60 years old, non-Hispanic White, single)*

*“Because I also wanted to learn what the study was about. I’m always in to learning something new.” (female, 40-50 years old, non-Hispanic Black/African American, single)*

Additionally, the women believed participation in the study provide the opportunity for self-reflection by allowing them to examine their life and recognize the areas that they would like to incorporate changes.

*“Reasons why I went into the study in the first place – to find myself, why am I doing this stuff to myself, you know?” (female, 50-60 years old, non-Hispanic White, single)*

*“I think it gave me time to reflect on the way I had been and where I’ll wind up at until where I am now. I wasn’t a drinker. I got into this relationship. He was a drinker, so just being a part, I started drinking.” (, female, 40-50 years old, non-Hispanic Black/African American, single)*

*“The way I was carrying my life, and I wanted my life to be better. That’s why I participated in the program.” (female, 50-60 years old, non-Hispanic White, single)*

#### Social

Many women reported social reasons for study participation, such as peer pressure and monetary compensation. Several women discussed learning about the study through a friend who was a past or current study participant.

*“I was talking to some of the other ladies, and I heard them talking amongst themselves trying to be confidential, and they were talking, “Girl, I’m going to this [name of clinical trial].” “Girl, they pay you to stop drinking.” (female, 50-60 years old, non-Hispanic White, single)*

*“Through a friend. She told me about it. She gave me the phone number to call, and I asked her what it was about. She said it was related to alcohol. She said she knew I drank, so I found out through a friend.” (female, 50-60 years old, non-Hispanic Black/African American, in a relationship)*

Also, many women reported being influenced or pressured by members of their social networks (e.g. family, friends) to participate in the study as a way to manage their problem drinking.

*“A friend told me that they got an alcohol study going on, and told me to go. She says if I didn’t go there, she’s going to take me there, either that or AA.” (female, 50-60 years old, non-Hispanic Black/African American, single)*

*“My sister. She just kept telling me, “Sis, I think you need to get into this program; it’s something good.” I said, “Girl, I don’t want to get in that. Don’t tell me anything about that.” She kept talking to me; “Sis, you need to get yourself together.” I said, “Okay, I’ll try it.” (female, 50-60 years old, non-Hispanic Black/African American, single)*

Though the majority of the women in this study were unemployed, participating in the study provided the opportunity to earn supplemental income.

*“I was motivated because I saw a lot of people going. Do you know what I mean? Now I got paid to go. I found myself when I got paid to go, I said, “God was doing for me what I couldn’t do for myself.” I used to pray about things that I wanted to stop doing.” (female, 50-60 years old, non-Hispanic black/African American, single)*

*“Just money because I am on a fixed income … ” (female, 50-60 years old, non-Hispanic Black/African American, single)*

*“Well, I had issues at the moment, I needed a couple of dollars too. So it was – they helped me and I helped them. I gave them what they needed and they gave me some money.” (female, 30-40 years old, non-Hispanic Black/African American, single)*

### Experience and retention in the WHAT-IF? Study

#### Biological

Women in the WHAT-IF? study were randomly assigned to receive either 50 mg of Naltrexone or placebo for 4 months. In the qualitative study, women discussed their experience with taking the study medication, although they were unaware of which study arm they were in. Many of the women discussed the effects of the study medication on their bodies, including any experienced adverse health effects.

*“The medication, it worked.” (female, 40–50 years old, non-Hispanic Black/African American, single)*

*“Excellent. It was actually because I didn’t have – there was no side effects. (female, 40–50 years old, Hispanic, single)*

*“The medication wasn’t a problem because – I think I became immune to taking meds, (laughs) I’m just going to say because I have to take meds, my little regiment that I have to take. The pills wasn’t a problem.” (female, 40–50 years old, non-Hispanic Black/African American, single)*

Among the women who did not like taking the study medication, reasons included experiencing adverse effects such as drowsiness, issues with their body, and perceived medication interactions.

*“It made me sleepy all the time. I would have to take it once a day so I would take it at night because it’s gonna’ put me to sleep. At first I started having these headaches from it, then I was nauseous but I got used to taking it.” (female, 40–50 years old, non-Hispanic Black/African American, single)*

*“Them things [pills] make me feel like my skin was on backwards.” (female, 50–60 years old, non-Hispanic Black/African American, single)*

*“As far as the pills are concerned, I was tired of taking those pills. It was toxic. Knowing that I had the virus I had eliminated a lot of toxins, so sometimes I wouldn’t actually take all the pills that they provided for me. Sometimes they would hit the commode, you know, because I was taking other HIV medications, high cholesterol, and so forth and so on. That’s toxic, you know, and I had to eliminate a lot of toxins from my body. After all, I was drinking and I wanted to get rid of that. With all those different types of pills, it’s like a bomb. It can blow with alcohol. I could blow up and not be anything and maybe get on dialysis or something, you know?” (female, 50–60 years old, non-Hispanic Black/African American, single)*

#### Psychological

During the qualitative interviews, the women reported positive and negative experiences in the WHAT-IF? study. Many of these experiences stemmed from the clinical trial study procedures (e.g. medication, lab work, study assessments) and the length of the study which was 7 months. While the majority of the women believed the study medication played a role in helping to reduce or quit their drinking.

*“Like I said, I don’t know if I was getting the real thing or the phony thing, but I think mentally, because I felt that it was helping me, I was actually helping myself.” (female, 50–60 years old, non-Hispanic Black/African American, single)*

*“Ah I liked it because it kept me aware it made me think you know you have to be careful now you taking these pills you don’t want the medication to react with the alcohol so the pills kind of kept me like ah ok now you don’t know what this stuff is you’re taking.” (female, 50–60 years old, non-Hispanic Black/African American, single)*

Moreover, some women discussed negative experiences with taking the study medication.

*“When I first started taking the medication, it was up and down with me – up and down. Some days, maybe at the end of the month, I forgot to take the medication. What I had to do was put it on an alcohol beverage; to say, “You need to take this. Maybe this will help you.” That’s basically how it helped me to remember to take the medication.” (female, 40–50 years old, non-Hispanic Black/African American, single)*

*“I didn’t like the medication.” (female, 40–50 years old, non-Hispanic Black/African American, single)*

*“The medication I really didn’t want to take it.” (female, 50–60 years old, non-Hispanic Black/African American, single)*

*“I have no complaints. They gave me that pill. I didn’t like that, but I don’t like taking medication. So it wasn’t just – even though I’m positive, I don’t like taking pills.” (female, 30–40 years old, non-Hispanic Black/African American, single)*

The majority of the women discussed negative experiences with having their blood drawn. For many of the women, their small veins made the procedure challenging.

*“I hate getting’ stuck because they can’t stick me one time they have to stick me more than once because I have these old baby veins. I have to always get stuck more than once, twice mostly more than twice.” (female, 40–50 years old, non-Hispanic Black/African American, single)*

*“Yeah, drawing blood. They had a heck of a time drawing my blood. I am a hard stick, figuring out these little thin, spider webby veins. They’re so tiny– it was just terrible. I had to drink a lot of water. One time they had to do me six times to get my blood.” (female, 50–60 years old, non-Hispanic White, single)*

While enrolled in the WHAT-IF? study, the women were required to complete computer assessments. During the assessments, women were prompted to answer questions about their past and current drinking. After the assessments, the women received feedback and information they could use to assist with making a behavioral change.

*“I like answering the different questions on the computer. I like when they asked me how long I had been going before I had a drink you know different questions they were asking me. I can’t remember per se you had them in steps.” (female, 50–60 years old, non-Hispanic Black/African American, single)*

*“The questions, they were personal questions. You know, what led me to drinking? Was it something? Was I depressed? Was I going through some type of changes? So that made me look at me. It was short, but it was interesting.” (female, 40–50 years old, Hispanic, in a relationship)*

*“That was fun to a certain extent. They were just so goddamn long. I don’t have patience that long. The only patience I have is sitting down reading the good books that I read.” (female, 50–60 years old, non-Hispanic Black/African American, single)*

Also, many of the women commented on the length of the WHAT-IF? Study which lasted for 7-months. The majority of the women believed the study length was appropriate and convenient.

*“Actually, it was a very convenient study so it wasn’t taking me out of nothing else I was really doing. I’d just go to the study, and then I’d go do whatever I was doing. It was very convenient. The time, I got to meet new people – you know, a couple people in there. It’s not far from my house, so it’s convenient.” (female, 30–40 years old, non-Hispanic Black/African American, single)*

*“It wasn’t a long, drawn out study. I mean because don’t wear me out …*” *(female, 30–40 years old, non-Hispanic Black/African American, single)*

#### Social

Many of the women reported the social interactions with the WHAT-IF? study staff impacted their experience and influenced their retention in the study. The women discussed their ability to talk freely and socialize with study staff. Also, multiple women praised the study staff for their professionalism, compassion, and respect that was displayed throughout the study and follow-up, respectively.

*My good experience is just sitting down and like what I’m doing with you now, but kind of different, just discussing my drinking, and how I drink, and how much I drink. Even though we’re talking about that, I was able to go outside of the boundaries a little bit and express my feelings or something personal, and the recruiter was open and there just to listen. That was good for me. (female, 40–50 years old, non-Hispanic Black/African American, single)*

*“I love the staff. The staff welcomed me. I felt comfortable and safe. I could talk to them about what’s going on with me. They were more like my listeners, not just only an employee, and they allowed me to be myself, and they treated me with respect, regardless of what my past was. I felt comfortable.” (female, 40–50 years old, non-Hispanic Black/African American, single)*

*“I like the people. They’ll pull you away. They draw you to them. They try to solve your problems. They try to keep you in firm. I just like it.” (female, 50–60 years old, non-Hispanic Black/African American, single)*

*Actually, it was the encouragement because I know that research is about we’re going to get this data, and we’re going to pay you, but staff members from the study that were actually caring. I noticed there were questions that were not written on the paper. So to me, that showed me there was some kind of feeling there of care, and they would listen to everything I had to say. It wasn’t a rush or anything like that. (female, 40–50 years old, Hispanic, single)*

However, some women were not as positive about their study interactions. As noted in the comment, even though they described a negative interaction, they did not find the study staff at fault.

*“Only sometime, when I felt they was in [a hurry] but I was in my own hurry. When you get there, you have things planned out. Here they are, running a little late, and so it was me. I was in my own hurry.” (female, 40–50 years old, non-Hispanic Black/African American, single)*

## Discussion

Women with HIV and hazardous drinking were asked to participate in qualitative interviews to better understand their reasons for participating in the WHAT-IF? study and to discuss their experience (positive or negative) in the study. In this study, the reasons for participation and their experience in the study were examined within the context of the biopsychosocial model.

For many women, participating in the study could help them quit or reduce their drinking to nonhazardous levels. The women discussed psychological reasons for study participation, such as the ability to gain knowledge or a greater understanding of the negative effects of hazardous drinking on HIV disease progression and the opportunity for self-reflection by examining their lives and recognizing the areas for behavioral change. This finding was consistent with previous research examining reasons for clinical trial participation among women, such as the opportunity for self-reflection or self-empowerment [16, 18]. Women in both studies believed their lives were out of control or needed services to help them regain control of their lives [16, 18]. Moreover, many of the women discussed social reasons for study participation, such as being influenced or pressured by members of their social network (e.g. family and friends). Besides, participation in the study allowed the women to earn supplemental incomes, as most were unemployed. While there were a few women who were employed and participated in the study, clinical trial planners may need to be considerate of the number of times participants may need to request off work to participate in the study.

Regarding clinical trial experience and study retention, the women discussed the factors (positive or negative) that contributed to their overall experience that may have contributed to the above satisfactory retention rates. During the interview, the women described their experience with taking the study medication (naltrexone or placebo) with most women reporting no adverse health effects. Among the women who did experience negative adverse effects, feelings of drowsiness and concerns about drug interactions were biological factors associated with their clinical trial experience. Several psychological factors (positive and negative) were associated with the clinical trial experience of women in the WHAT-IF? study, such as thoughts and feelings toward study procedures (e.g. study medication, lab work, and study assessments) and the length of the study (7 months). Though the women discussed their displeasure with some of the aforementioned study procedures, these experiences were not strong enough to deter the women from completing the study. In order to be responsive to participants’ complaints, future research could strive to acknowledge that their concerns cannot really be addressed without changing the nature of the study (e.g. bloodwork is necessary) or providing accommodations to participants (e.g. giving participants water in advance of bloodwork). Thus, after examining women’s experience in the WHAT-IF? study, the consensus was overwhelmingly positive. This study reported similar findings among study participants who discussed how positive interactions with study staff were instrumental to their success in the study [26, 41].

Moreover, determining the most effective strategies for the recruitment and retention of women and minorities in alcohol intervention research remain an understudied area [48]. A contributing factor to this disparity is medical mistrust. African Americans’ medical mistrust has stemmed from prior historical institutional discrimination, first-hand experiences in healthcare, historical segregation, as well as unethical medical treatment and care [2]. Pursing this further, HIV-related mistrust among African Americans’ include conspiracy theories about HIV’s origin and treatment [2]. To date, the WHAT-IF? study was the first clinical trial to examine pharmacotherapy for a reduction in hazardous drinking among a sample of women with HIV [8]. While the majority of women in the WHAT-IF? study were Black/African-American, the inclusion of minority women in alcohol intervention research is scarce [8, 47, 48]. To address this shortcoming, the successful recruitment of women and minorities may require additional planning, timelines, and budget requirements [10, 34, 47, 48]. Likewise, reducing barriers to study participation among women, such as economic expenses, individual challenges, and general reluctance could increase treatment-seeking practices [10, 22, 31, 33, 43]. In addition, understanding different groups’ particular socio-economic circumstances could ensure that people understand that women and individuals from different ethnic minority groups have particular health conditions/health-seeking challenges primarily because of their socio-economic circumstances and not because of their ethnicity [38, 40, 42]. Besides, developing a rapport with communities of color, using their networks for advertisement, and including some of the participants as peer researchers and co-authors in the study could increase study participation, particularly among Black/African-Americans [27, 43].

Some limitations of the study warrant mention. First, only women with HIV and hazardous drinking who completed the WHAT-IF? study was asked to participate in the qualitative interviews. The Miami-based research coordinator recruited the women using convenience and purposeful sampling. In addition, many of the participants mentioned that their family members and friends were influential in their decisions to seek care. While none of the participants mentioned being coerced to participate, it is important to note the influence of social networks on drinking behaviors as well as study participation [12, 13, 28]. Also, the majority of the participants in both studies were Black/African-American women with HIV and hazardous drinking. Thus, the results may not be generalizable to the population of hazardous drinking women with HIV. Moreover, our characterizations of reasons for study participation and their experiences are based on participant narrative accounts of being enrolled in the WHAT-IF? study. Due to the self-reported nature of data collected during the qualitative interviews, we are unable to determine the accuracy of the information. While the women provided documentation to confirm their HIV status, self-report data were used to determine their hazardous drinking status. As a result, we are unable to confirm the accuracy of the reported data. Moreover, there was variability in the time between the women completing the WHAT-IF? study and participating in the qualitative interviews. In this study, some of the women completed the interviews within a few months of completing the WHAT-IF? study, whereas others completed the WHAT-IF? study up to 2 years earlier. Because of the potential recall bias, the findings of this study are the women’s’ narrative account of their reasons for participating in the WHAT-IF? study and their experience.

## Conclusion

Recruiting and retaining women with HIV in alcohol intervention research remains a challenge. Conducting qualitative research (particularly in underserved, disadvantaged groups) could provide critical data on the perceptions, cultural relevancy, acceptability, and salience of specific aspects of intervention approaches of the randomized clinical trial that are acceptable and not acceptable to these populations. Findings from this study suggest that women with HIV who are hazardous drinkers may benefit from participating in research studies that could help them to reduce or quit their drinking, increase their knowledge about specific behavior changes, and earn monetary compensation. Moreover, positive staff interactions may also be instrumental in retaining minority women in alcohol intervention research. Besides, findings may be useful for providing a basic understanding of the factors associated with recruitment, experience, and retention of women in alcohol intervention research.

## Data Availability

The datasets used and/or analyzed during the current study are available from the corresponding author on reasonable request.

## Abbreviations

NIH: National Institute of Health
WHAT-IF?: Will Having Alcohol Treatment Improve my Functioning
QRC: Qualitative Research Colloquium
CRC: Clinical Research Building
BMRC: Behavioral Medical Research Center
HIPAA: Health Insurance and Portability and Accountability Act
